# Priority Analysis of Educational Needs of Forest Healing Instructors Related to Programs for Cancer Survivors: Using Borich Needs Assessment and the Locus for Focus Model

**DOI:** 10.3390/ijerph19095376

**Published:** 2022-04-28

**Authors:** Kwang-Hi Park, Min Kyung Song

**Affiliations:** 1 College of Nursing, Gachon University, Incheon 21936, Korea; parkkh@gachon.ac.kr; 2 Department of Nursing, College of Medicine, University of Ulsan, Ulsan 44610, Korea

**Keywords:** cancer survivors, forest healing instructors, needs assessment

## Abstract

This study aimed to identify the priority of educational needs to strengthen the competency of forest healing instructors who operate forest healing programs for cancer survivors. A survey was conducted with 91 forest healing instructors using a questionnaire. The average perceived importance of the forest healing program for cancer survivors of forest healing instructors was higher compared to the average knowledge of the forest healing instructors. The Borich needs assessment model showed the highest educational need in the domains of “Cancer survivors’ overview” and “Health status screening method”. In addition, deriving the priority of educational needs using the Borich needs assessment model and the focus trajectory model revealed that the highest priority was for “Health status screening method” and “Effective communication with cancer survivors”. These results can be used as basic data for developing a forest healing instructors training program for cancer survivors that reflects the instructors’ needs and the characteristics of cancer survivors.

## 1. Introduction

With the increase in cancer incidence (the number one cause of death in Korea) and advances in medical technology, the survival rate of cancer is increasing because of early detection and improvement of treatment [1]. In the case of Korea, the 5-year relative survival rate of cancer patients diagnosed in a recent 5-year period (2015–2019) was 70.7%, which was a 5.2% improvement, compared to the 65.5% survival rate in 2006–2010 [2]. Cancer is recognized as a chronic disease that requires long-term management due to the improvement in survival rate [3,4]. Cancer survivors can experience acute, chronic, physical, and psychosocial health problems due to cancer treatment and recovery [5]. Many cancer survivors also experience mental health challenges, such as fear of cancer recurrence, anxiety, and depression [6]. Therefore, interventions are needed to help cancer survivors alleviate these problems.

As interest in health increases, efforts to restore health and improve quality of life in nature and forests have increased, along with increased interest in forest healing. Forest healing involves immune-strengthening and health-promoting activities, which utilize a variety of elements of the forest, including scents and scenic views [7]. In Korea, various forest welfare services such as forest Taegyo (prenatal education), children’s forest experience, and forest therapy are provided by the Korea Forest Welfare Institute, which creates and provides services such as forest culture, recreation, forest healing, and education based on forests [8]. In the past 10 years, various studies have reported that time spent in nature has a positive effect on mental and psychological well-being [9,10,11], and it has been reported that forest healing programs are effective in improving well-being and psychological problems among all ages [12,13]. In addition, a systematic review regarding the effects of forest healing activities on adult patients with various diseases reported positive effects not only in physiological indicators such as blood pressure, NK cell, and perforin, but also in psychosocial aspects [14]. For cancer survivors, forests are the best recovery environment for reducing stress through various environmental factors [15].

As the number of spaces available for forest healing increases and the demand for forest healing rises, the need for quality forest healing programs is also increasing [16,17]. In Korea, forest healing instructors, who plan and develop forest healing programs tailored to each target using forests so that visitors can experience forest healing activities effectively, are nurtured. The forest healing program is dependent on the individual competency of the forest healing instructor who directly develops and intervenes in the program. Forest healing factors, programs, instructors, and facilities have been mentioned as important factors considered by participants in a forest healing program [18]. In forest healing activities, the higher the social and emotional support of the program participants, the higher the level of continuity and motivation to participate, which can bring a greater healing effect [19]. The role of forest healing instructors in carrying out forest healing programs is also considered important [20]. Therefore, to improve the quality of forest healing programs, it is necessary to strengthen the capabilities of forest healing instructors.

Educational need is the discrepancy or gap between “what is” or the current state for interest groups and situations and “what should be” or the desired state [21]. Analysis of the knowledge level (current status) and importance (desired status) of forest healing instructors who operate programs for cancer survivors at the forest healing site and determining priorities play an important role in meeting their educational needs. However, to the best of our knowledge, no study has analyzed the educational needs of forest healing instructors for cancer survivors. Therefore, in this study, the knowledge and importance level of forest healing instructors who operate forest healing programs targeting cancer survivors are identified, and based on this, educational needs are clarified. The specific objectives of this study were as follows: (i) determining the knowledge level and importance of forest healing instructors for programs for cancer survivors, and (ii) identifying forest healing instructors’ priority of educational needs to develop programs for cancer survivors, using the Borich needs assessment model [21] and the Locus for Focus Model [22]. Through this study, it will be possible to improve forest healing instructors’ ability to conduct programs for cancer survivors and to develop more realistic and applicable educational programs.

## 2. Materials and Methods

### 2.1. Study Design and Participants

This study is a descriptive research study to identify the priority of educational needs of forest healing instructors for forest healing programs targeting cancer survivors. The participants were forest healing instructors with experience in forest healing programs. The exclusion criterion was having no experience in providing forest healing programs. The sample size was calculated using G*Power 3.1.9 (University of Dusseldorf, Dusseldorf, Germany). In the paired-sample *t*-test, the minimum sample size was 90 patients with an effect size of 0.30 [23], a significance level of 0.05, and a statistical power of 0.80. Considering the 10% dropout rate, 105 forest healing instructors were recruited. To block the possibility of multiple responses, it was checked whether the cell phone number, which is personally identifiable information, was duplicated.

### 2.2. Ethical Approval

This study was conducted with the ethical approval of the Institutional Review Board (IRB No. blinded). The collected data were assigned a random ID so that participants could not be identified and stored in an encrypted file to keep the subject’s personal information confidential. The mobile phone number written in the questionnaire response data was for the payment of the fee (mobile gift certificate), and it was permanently deleted after the payment was completed.

### 2.3. Measures and Data Collection

#### 2.3.1. Questionnaire

The questionnaire consisted of items such as participants’ demographic characteristics, experience with forest healing programs for cancer survivors, knowledge level, and perceived importance of forest healing programs for cancer survivors.

Cancer survivors were defined in this study as “cancer patients who have completed major treatment (surgery, chemotherapy, radiation therapy) for the purpose of cure after cancer diagnosis”. The questions about the knowledge level and perceived importance of forest healing programs for cancer survivors included 24 items on five domains: cancer survivors’ overview, cancer survivors’ emotions, and cancer survivors’ physical, spiritual, family, and functional problems. Each item of knowledge level was composed of a 4-point Likert scale (1: not aware at all to 4: very well aware) and each item of perceived importance consisted of a 4-point Likert scale (1: not important at all to 4: very important).

The validity of the contents of the questionnaire was evaluated through the recommendations of a group of 10 experts (4 nursing professors, 5 forest healing instructors, and 1 researcher at the National Institute of Forest Science). The content validity index of the questionnaire items was 0.8 or higher, and all items were retained. In addition, the contents of some items were supplemented to increase the understanding of the topic of the question and to facilitate the flow of sentences. The reliability of the knowledge level was 0.960, and the reliability of perceived importance was 0.975 (Supplementary Materials File S1).

#### 2.3.2. Data Collection

Forest healing instructors were recruited through an online community for such instructors. One researcher with a Forest Healing Instructor’s license was allowed to explain the research details to the president of the Forest Healing Instructor’s Association and post a recruitment notice with a URL to the survey. Data collection was conducted through an online survey (Google Forms) from 24 May to 11 June 2021. At the start of the online survey, explanations including the purpose and method of the study were provided, and consent was obtained for participation in the study. Participants were guaranteed the right to voluntarily participate in the study and their privacy. To prevent data omission, all questions in the online survey were set so that the respondent had to answer the previous question before moving on to the next question. The average time to complete the questionnaire was about 20 min, ranging from 15 to 25 min. Finally, 91 participants were used for the analysis, excluding 14 persons without forest healing instructor experience.

### 2.4. Statistical Analysis

The collected data were analyzed using the SPSS 25.0 program (IBM Corp, Armonk, NY, USA). Standard descriptive statistics were used for general characteristics, experience with forest healing programs for cancer survivors, and knowledge level and perceived importance of forest healing programs for cancer survivors. Differences in knowledge level and perceived importance according to general characteristics were analyzed using an independent *t*-test and analysis of variance (ANOVA), and a paired *t*-test was conducted to verify differences in knowledge level and perceived importance.

To analyze the educational needs for forest healing programs for cancer survivors, the Borich needs assessment model and the Locus for Focus Model were used for analysis. The Borich needs assessment formula is

$$\mathrm{Needs}=\frac{\sum \left(RL-PL\right)\times \bar{RL}}{N}$$

*RL*: required level (perceived importance), *PL*: present level (knowledge level), $\bar{RL}$: average of the required level, *N*: total number of cases

According to this formula, a higher required level (*RL*) and a lower present level (*PL*) indicate higher requirements. The Borich needs assessment model has the advantage that a rational and valid educational needs analysis is possible by applying weights to required level beyond the present level-required level gap [23]. Borich needs assessment has been used to evaluate the current performance and future needs of forest interpreters, teachers, parents, nurses, etc. [23,24,25].

The Locus for Focus Model showed the priority of educational needs on an *x*-*y* plane. The line parallel to the *x*-axis indicates the value of the discrepancy between perceived importance and knowledge level, and the line parallel to the *y*-axis indicates the average of perceived importance. Therefore, the first quadrant (high discrepancy/high importance, HH) represents the area with the highest priority of education demand among the four quadrants that is higher than the average importance and larger than the average deviation. The second quadrant (high discrepancy/low importance, HL) represents lower-than-average importance but a higher degree of discrepancy than the average. This indicates the second highest priority because it is necessary to understand the low importance and increase the performance.

The highest priority according to Borich needs assessment was determined based on the number of items belonging to the first quadrant (HH quadrant) in the Locus for Focus Model, and the highest priority was determined by checking the redundancy between the items. To understand the importance perceived as low in the second quadrant (HL quadrant) of the Locus for Focus Model and increase performance, the HL area in the second quadrant was selected as the next priority, and items that overlapped with the Borich needs assessment were determined as the next priority.

## 3. Results

### 3.1. Participant Characteristics

The average age of the participants was 55.7 years (SD = 8.1); overall, 68.1% were women, and 52.7% had a level of education above graduate school. The main majors of the participants were forest-related fields (49.5%), followed by nursing, public health, and medicine (25.3%) and others (25.3%). The average experience of forest healing instructors was 30.7 months (SD = 25.0). Currently active participants amounted to 61.5%, and 39.3% reported that they were active in urban forests/parks.

A total of 58.2% participants reporting having no experience in operating a program for cancer survivors, and in the case of no experience in the program, the degree of difficulty was 2.74 points, which was statistically significantly higher than in the case of having experience (t(87) = 4.57, *p* < 0.001, effect size d = 0.981). The forest healing instructors who had experience in operating programs for cancer survivors answered that the difficulties during the program were sensitive participants (38.0%), fear of an emergency (26.0%), and lack of knowledge (24.0%). Meanwhile, the expected difficulties of forest healing instructors without experience in operating programs for cancer survivors were similar, but fear of an emergency was the highest at 33.0%. A total of 98.9% participants answered that they were willing to participate in educational programs on forest healing programs for cancer survivors (Table 1).

### 3.2. Forest Healing Instructors’ Knowledge Level and Perceived Importance of Forest Healing Programs for Cancer Survivors

The overall knowledge level of the forest healing instructors on cancer survivors was an average of 2.78 ± 0.66 out of 4 points, and emotional and spiritual problems were the highest with 2.93. The domain with the lowest knowledge level was cancer survivors’ overview (2.42 ± 0.60). Regarding each item, “Change in appearance (hair loss, skin color)” showed the highest and “Health status screening method” showed the lowest scores. The overall perceived importance of forest healing programs for cancer survivors averaged 3.24 ± 0.59 out of 4 points, and spiritual problems were the highest at 3.40 ± 0.56. The domain with the lowest educational needs was functional problems, with a score of 3.14 ± 0.54. Regarding each item, “Effective communication with cancer survivors” showed the highest and “Status for cancer survivor” showed the lowest scores (Table 2).

### 3.3. Differences in Knowledge Level and Perceived Importance of Cancer Survivors according to General Characteristics of Forest Healing Instructors

Analysis of the differences in knowledge level and perceived importance of cancer survivors according to the general characteristics of forest healing instructors revealed that there was no difference according to gender, education level, major, current activity, or experience in operating the cancer survivor program (Table 2).

### 3.4. Borich Needs Assessment Analysis of Educational Needs for Forest Healing Programs for Cancer Survivors

The average educational need among forest healing instructors regarding forest healing programs for cancer survivors, according to the Borich needs assessment model, was 1.49. Among the domains, “cancer survivors’ overview” showed the highest educational need, while “functional problems” had the lowest educational need. Items 4 (health status screening method), 3 (effective communication with cancer survivors), and 1 (status for cancer survivors) had the highest educational need (Table 3).

### 3.5. The Locus for Focus Model of Educational Needs for Forest Healing Programs for Cancer Survivors

**Figure 1:**
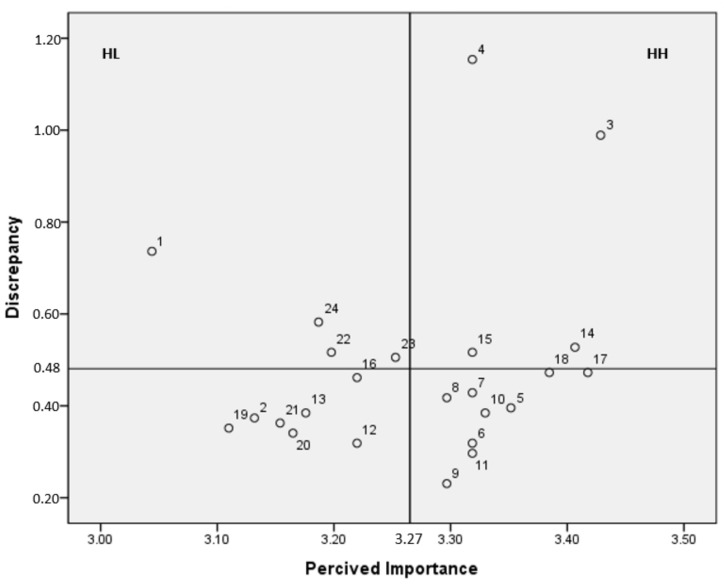
Visualization of priority of forest healing instructors’ educational needs for forest healing programs for cancer survivors using the Locus for Focus Model. Refer to the Table 3 for items 1 to 24.

The result of visualizing the priority using the Locus for Focus Model is shown in Figure 1. The first quadrant represented the highest priority, and 4 items (4. health status screening method, 3. effective communication with cancer survivors, 14. pain, and 15. sleep disorder) were included in the first quadrant (Figure 1).

### 3.6. Priority of Educational Needs for Forest Healing Programs for Cancer Survivors

This section involves checking for redundancy of items proposed as a high priority according to the Borich needs assessment model and the Locus for Focus Model. The items determined to have the highest priority were 4. health status screening method and 3. effective communication with cancer survivors. The items determined to have the second highest priority were 1. status for cancer survivors, 24. relationship with parents, 14. pain, and 15. sleep disorder (Table 4).

## 4. Discussion

This study attempted to identify the priority of educational needs to strengthen the competency of forest healing instructors who operate forest healing programs for cancer survivors. Forest healing-related research has mainly focused on participants who use the program, and there is a lack of research on forest healing instructors who operate the program. For more efficient program operation, it is necessary to improve the knowledge and quality of program providers. Optimally effective program operation will be possible if a forest healing program is developed and operated to improve the health of the increasing number of cancer survivors by integrating the needs of forest healers who provide the program.

There was no difference in the knowledge level of forest healing instructors according to general characteristics. Of the forest healing instructors who participated in this study, only 25.3% of them majored in nursing/medicine/health care, and most of them were non-health workers. However, there was no difference in the knowledge level of forest healing instructors according to their majors, and this is thought to be the effect of the fact that 52.7% of the participants received above graduate school education. Previous research in which more than 90% of forest healing instructors had a college degree or higher can support this [26]. This is believed to be because forest healing instructors are increasing their professionalism by acquiring medical knowledge and skills during training [27], and their interest in health is also high. In addition, their average age is generally high (55.7 years); therefore, forest healing instructors may have met cancer survivors in their acquaintances and family members.

In the case of program operation, the degree of difficulty was relatively higher for those who had no experience in operating a program for cancer survivors. Although the detail ratio is somewhat different, the difficulty was reported to be because of fear of an emergency, participants’ sensitivity, or lack of knowledge. This is thought to be related to fearfulness of proceeding with the program due to prejudice and stigma [28] about cancer, which is a negative image related to physical weakness and death, even though the subjects are cancer survivors after active treatment has been completed.

The domain with the highest priority of educational needs of forest healing instructors for cancer survivor programs was “Cancer survivors’ overview”. This is because the knowledge level was lower than the average for all items, and the domain was considered to have an average level of perceived importance. Even in the case of forest healing instructors who are skilled in forest healing programs through training process and practice, if the target group is a special group, it may be difficult to engage in professional healing activities if they do not have an understanding of the participants [29]. In particular, forest healing instructors in charge of forest healing programs for cancer survivors require additional competency compared to forest healing instructors who operate general forest healing programs, because of the characteristics of cancer survivors [29,30]. Therefore, when developing forest healing programs for cancer survivors in the future, education about the cancer survivors’ overview is necessary, and periodic education is necessary. In the cancer survivors’ overview domain, the top two items that required education were “Health screening method” and “Effective communication with cancer survivors”. Cancer survivors have health problems such as late-stage side effects, increased risk of chronic diseases, anxiety, and depression [31]. In addition, as side effects of treatment appear differently depending on the type of cancer [31], the application method and points to be considered differ depending on survivors’ characteristics when providing programs such as exercise. There was a high demand for a health status screening method to prevent emergencies and to check the participant’s health condition because of the nature of the forest healing program, which involves taking survivors to the forest. In addition, the demand for communication was also believed to be high owing to the negative image [28] and psychological barriers specific to cancer. Forest healing instructors who want to heal the target by using elements in the forest are also thought to have needs for effective communication. In fact, cancer survivors are known to experience psychological distress such as depression and anxiety [28,32]. Effective communication is important because it plays an important role in the emotional health of cancer survivors [26]. Therefore, programs for cancer survivors should be led by forest healing instructors with specialized knowledge [30]. It is necessary to develop an education program for forest healing instructors including education on communication, accompanied by continuous education, in the future.

In cancer survivors, sensitive reactions and stress are induced in all aspects of daily life [15]. Stress relief and positive emotional changes in cancer survivors improve self-esteem and give meaning to life [15]. The forest healing program is a good recovery environment to reduce these stresses by using various environmental factors of the forest [15,33,34]. In order to improve the health of cancer survivors by utilizing the environmental factors of the forest, continuous specialized education is required to improve the professional competency of forest healing instructors. For forest healing to be activated as one of the integrated interventions for supporting cancer survivors, practical education on the characteristics of cancer survivors will be required for forest healing instructors. In particular, health professionals should be included when developing forest healing programs for people with diseases such as cancer survivors or providing training for forest healing instructors for such programs to strengthen the capabilities of forest healing instructors. Strengthening the competence of forest healing instructors through more professional health-related education programs will enable them to operate the program confidently, which will eventually improve cancer survivor’s outcomes and satisfaction. In addition, professional health care training for such disease should be included in continuing education, and furthermore, it is necessary to establish corresponding policies to enable forest healing instructors who complete appropriate hours of training to operate such healing programs.

A limitation of this study is that the results of this study cannot be generalized because the sample of forest healing instructors who participated in the study was small. Therefore, in follow-up studies, it will be necessary to collect sufficient samples to evaluate the competency and professionalism of forest healing instructors for cancer patient program operation and to confirm educational needs. In addition, it will be necessary to confirm additional educational requirements through qualitative research involving individual interviews or focus group interviews. Nevertheless, this study is meaningful in that it confirmed the educational needs of forest healing instructors for cancer survivors and helped build basic data that can be expected to provide high-quality services and disseminate services through strengthening the competency of forest healing instructors. Based on this, if a forest healing instructor training program is developed and services that reflect the characteristics of cancer survivors are applied, the service availability for consumers will be improved. This study can be used as important basic data for developing educational materials and guidelines for nurturing forest healing leaders who can operate effective forest healing programs for cancer survivors.

## 5. Conclusions

A forest healing instructor is an expert who conducts forest healing programs by acquiring specialized knowledge in forestry and health care. However, for cancer survivors who have health problems such as late-stage side effects, increased risk of chronic diseases, anxiety, and depression, forest healing instructors with the capacity to conduct programs that reflect their specificities are needed. For this purpose, it is necessary to develop practical education programs and continuous education on the characteristics of cancer survivors for forest healing instructors.

## Figures and Tables

**Table 1:** Participants’ characteristics and cancer survivor program operation experience.

Variables	Categories	n (%) M ± SD	t (*p*)
Age		55.7 ± 8.1	
Gender	Female	62 (68.1)	
	Male	29 (31.9)	
Education level	High school or university graduate	43 (47.3)	
	≥Graduate school	48 (52.7)	
Major	Forest-related fields	45 (49.5)	
	Nursing/Health Science/Medicine	23 (25.3)	
	Others	23 (25.3)	
Working period (month)		30.7 ± 25.0	
Currently active	Yes	56 (61.5)	
	No	35 (38.5)	
Currently active facility	Natural recreation forest	8 (14.3)	
	Healing forest	20 (35.7)	
	Urban forest/park	22 (39.3)	
	Others	6 (10.7)	
Program operation experience for cancer survivors	Yes	38 (41.8)	
	No	53 (58.2)	
Degree of difficulty in program operation	Had experience	2.05 ± 0.70	−4.57 (<0.001)
	No experience	2.74 ± 0.71	
Difficulty reason ^1^ (Had experience)	Fear of an emergency	13 (26.0)	
	Fear that the participant is sensitive	19 (38.0)	
	Fear that the participant’s health may deteriorate	3 (6.0)	
	Hard to talk	2 (4.0)	
	Lack of knowledge	12 (24.0)	
	Others	1 (2.0)	
Difficulty reason ^1^ (No experience)	Fear of an emergency	29 (33.3)	
	Fear that the participant is sensitive	27 (31.0)	
	Fear that the participant’s health may deteriorate	8 (9.2)	
	Hard to talk	1 (1.1)	
	Lack of knowledge	21 (24.1)	
	Others	1 (1.1)	
Intention to participate in education	Yes	90 (98.9)	
	No	1 (1.1)	

^1^ multiple responses.

**Table 2:** Differences in knowledge level and perceived importance of forest healing programs for cancer survivors according to participants’ characteristics and cancer survivor program operation experience.

Variables	Categories	Knowledge Level		Perceived Importance	
		M ± SD	T, F (*p*)	M ± SD	T, F (*p*)
Gender	Female	2.78 ± 0.43	−0.21 (0.837)	3.31 ± 0.37	1.28 (0.205)
	Male	2.80 ± 0.53		3.18 ± 0.57	
Education level	High school or university graduate	2.69 ± 0.50	−1.89 (0.062)	3.19 ± 0.49	−1.54 (0.128)
	≥Graduate school	2.87 ± 0.40		3.33 ± 0.40	
Major	Forest-related fields	2.80 ± 0.51	1.40 (0.252)	3.28 ± 0.52	0.78 (0.463)
	Nursing/Public Health/Medicine	2.87 ± 0.33		3.33 ± 0.39	
	Others	2.66 ± 0.44		3.17 ± 0.32	
Currently active	Yes	2.85 ± 0.46	1.75 (0.084)	3.27 ± 0.37	0.11 (0.909)
	No	2.68 ± 0.44		3.26 ± 0.55	
Currently active facility	Natural recreation forest	2.89 ± 0.53	0.47 (0.707)	3.21 ± 0.30	1.03 (0.388)
	Healing forest	2.76 ± 0.54		3.21 ± 0.35	
	Urban forest/park	2.92 ± 0.39		3.38 ± 0.41	
	Others	2.82 ± 0.33		3.15 ± 0.39	
Program operation experience for cancer survivors	Yes	2.70 ± 0.51	−1.49 (0.140)	3.24 ± 0.53	−0.47 (0.639)
	No	2.84 ± 0.41		3.28 ± 0.38	

**Table 3:** Results of paired *t*-test and Borich needs assessment model to examine forest healing instructors’ educational needs regarding forest healing program for cancer survivors.

Domain	Items	Knowledge Level M ± SD	Perceived Importance M ± SD	Mean Difference M ± SD	T (*p)*	Borich Needs Assessment	Ranks
1. Cancer survivors’ overview		2.42 ± 0.60	3.23 ± 0.54	0.81 ± 0.68	11.47 (<0.001)	2.62	1
	1. Status for cancer survivors	2.31 ± 0.78	3.04 ± 0.54	0.74 ± 0.89	7.87 (<0.001)	2.24	3
	2. Cancer treatment (surgery, chemotherapy, radiation therapy, etc.)	2.76 ± 0.74	3.13 ± 0.60	0.37 ± 0.80	4.47 (<0.001)	1.17	17
	3. Effective communication with cancer survivors	2.44 ± 0.73	3.43 ± 0.67	0.99 ± 0.86	10.94 (<0.001)	3.39	2
	4. Health status screening method	2.16 ± 0.69	3.32 ± 0.66	1.15 ± 0.91	12.15 (<0.001)	3.83	1
2. Emotional problems		2.93 ± 0.44	3.32 ± 0.52	0.39 ± 0.58	6.44 (<0.001)	1.29	4
	5. Depression/sadness	2.96 ± 0.47	3.35 ± 0.57	0.40 ± 0.61	6.16 (<0.001)	1.33	14
	6. Fear/worry	3.00 ± 0.47	3.32 ± 0.53	0.32 ± 0.58	5.29 (<0.001)	1.06	21
	7. Nervousness/irritability	2.89 ± 0.53	3.32 ± 0.56	0.43 ± 0.72	5.70 (<0.001)	1.42	12
	8. Loss of motivation	2.88 ± 0.51	3.30 ± 0.55	0.42 ± 0.65	6.12 (<0.001)	1.38	13
3. Physical problems		2.90 ± 0.51	3.29 ± 0.48	0.39 ± 0.61	6.12 (<0.001)	1.28	5
	9. Change in appearance (hair loss, skin color)	3.07 ± 0.49	3.30 ± 0.53	0.23 ± 0.60	3.68 (<0.001)	0.76	24
	10. Diet (weight/intake change)	2.95 ± 0.54	3.33 ± 0.56	0.38 ± 0.68	5.40 (<0.001)	1.28	15
	11. Fatigue	3.02 ± 0.52	3.32 ± 0.53	0.30 ± 0.59	4.82 (<0.001)	0.98	23
	12. Indigestion (nausea)	2.90 ± 0.60	3.22 ± 0.55	0.32 ± 0.70	4.36 (<0.001)	1.03	22
	13. Memory/reduced concentration	2.79 ± 0.68	3.18 ± 0.51	0.38 ± 0.76	4.85 (<0.001)	1.22	16
	14. Pain	2.88 ± 0.70	3.41 ± 0.56	0.53 ± 0.85	5.94 (<0.001)	1.80	5
	15. Sleep disorder	2.80 ± 0.64	3.32 ± 0.53	0.52 ± 0.78	6.32 (<0.001)	1.71	6
	16. Numbness of limbs	2.76 ± 0.69	3.22 ± 0.49	0.46 ± 0.76	5.76 (<0.001)	1.49	11
4. Spiritual problems		2.93 ± 0.57	3.40 ± 0.56	0.47 ± 0.71	6.33 (<0.001)	1.60	3
	17. Anxiety about recurrence/death	2.95 ± 0.60	3.42 ± 0.58	0.47 ± 0.75	6.01 (<0.001)	1.61	9
	18. Worries about the meaning of life	2.91 ± 0.61	3.38 ± 0.57	0.47 ± 0.75	6.01 (<0.001)	1.60	10
5. Functional problems		2.79 ± 0.62	3.14 ± 0.54	0.35 ± 0.69	4.87 (<0.001)	1.10	6
	19. Raising children	2.76 ± 0.64	3.11 ± 0.55	0.35 ± 0.72	4.65 (<0.001)	1.09	19
	20. Economic problems	2.82 ± 0.66	3.16 ± 0.54	0.34 ± 0.76	4.26 (<0.001)	1.08	20
	21. Work/school	2.79 ± 0.66	3.15 ± 0.61	0.36 ± 0.75	4.59 (<0.001)	1.14	18
6. Family problems		2.68 ± 0.68	3.21 ± 0.52	0.53 ± 0.79	6.42 (<0.001)	1.70	2
	22. Problems with children	2.68 ± 0.68	3.20 ± 0.52	0.52 ± 0.81	6.10 (<0.001)	1.65	7
	23. Relationship with spouse	2.75 ± 0.74	3.25 ± 0.57	0.51 ± 0.86	5.60 (<0.001)	1.64	8
	24. Relationship with parents	2.60 ± 0.71	3.19 ± 0.54	0.58 ± 0.84	6.58 (<0.001)	1.86	4
Total		2.78 ± 0.46	3.27 ± 0.45	0.48 ± 0.53	8.69 (<0.001)	1.49	

**Table 4:** Needs of forest healing instructors according to Borich needs assessment model and the Locus for Focus Model.

Domain	Contents	Rank (Borich Needs Assessment)	Quadrant (Locus for Focus Model)	High Priority
1	4. Health status screening method	1	Ⅰ	1
1	3. Effective communication with cancer survivors	2	Ⅰ	1
1	1. Status for cancer survivors	3	Ⅱ	2
6	24. Relationship with parents	4	Ⅱ	2
3	14. Pain	5	Ⅰ	2
3	15. Sleep disorder	6	Ⅰ	2

## Data Availability

The data presented in this study are available on request from the corresponding author.

## Supplementary Materials

The following supporting information can be downloaded at: https://www.mdpi.com/article/10.3390/ijerph19095376/s1, File S1: Questionnaire on priority analysis of educational needs of forest healing instructors related to programs for cancer survivors: using Borich needs assessment and the Locus for Focus Model.
