# Supplementary material for: Priority Analysis of Educational Needs of Forest Healing Instructors Related to Programs for Cancer Survivors: Using Borich Needs Assessment and the Locus for Focus Model
Source: Int J Environ Res Public Health. 2022 Apr 28;19(9):5376. doi: 10.3390/ijerph19095376 (PMC9103931; doi:10.3390/ijerph19095376)
Supplement: Supplementary file 1 [file ijerph-19-05376-s001.zip › ijerph-1683697-supplementary.pdf]

Questionnaire on priority analysis of educational needs of forest healing instructors related to programs for cancer survivors: using Borich needs assessment and The Locus for Focus Model

[Consent]

Welcome. Thank you for taking part in this study. This study examines the e**ducational** n**eeds of** f**orest healing instructors related to programs for cancer survivors**. You may be asked to respond to some questionnaires related to your personal information.
This survey may take **about 10 minutes to complete**, but the exact time depends on your individual performance.

Your participation is voluntary. **Your responses will remain confidential and will only be treated in an aggregate fashion**. The results of the survey may be published. You have the right to withdraw from the study at any time. If you agree to participate in the survey, please click the consent form. Consent is implied with the submission of the survey. Again, we appreciate your participation.

Section I. Demographics

1. What is your age? (   )

2. What is your gender?

① Male ② Female

3. What is the highest level of education?

① No formal education ② Elementary school ③ Middle school

④ High school ⑤ College/University ⑥ Graduate school

4 . What is your major?

① Forest-related fields ② Nursing/ Medicine/Health care ③ Others

5. What kinds of certifications do you have? (Multiple responses possible)

① Forest Healing Instructor (Level 1) ② Forest Healing Instructor(Level 2) ③ Forest Commentator

④ Infant Forest Instructor ⑤ Forest Trail Instructor ⑥ Others

6. How long have you worked as a forest healing instructor? (months)

7. Are you currently working as a forest healer?

① Yes (→ go to Q 8) ② No (→ go to Q 9)

8. What facility are you working at?

① Natural recreation forest ② Healing forest ③ Urban forest/park ④ Others

9. What was the last facility you worked for?

① Natural recreation forest ② Healing forest ③ Urban forest/park ④ Others

Section II. Program operation experience for cancer survivors

2-1. Do you have experience running programs for cancer survivors?

① Yes (→ go to Q 2-2) ② No (→ go to Q 2-4)

2-2. To what extent did you experience difficulties while running the program for cancer survivors?

① Not difficult at all ② A little difficult ③ Somewhat difficult ④ Very difficult

2-3. What difficulties did you experience while running the program for cancer survivors?
(Multiple responses possible)

① Fear of emergency

② Fear that the participant is sensitive

③ Fear that the participant's health may deteriorate

④ Hard to talk

⑤ Lack of knowledge

⑥ Others

2-4. How worried are you if you are running a program for cancer survivors?

① Not difficult at all ② A little difficult ③ Somewhat difficult ④ Very difficult

2-5. What difficulties do you think there will be if you proceed with the program for cancer survivors?

① Fear of emergency

② Fear that the participant is sensitive

③ Fear that the participant's health may deteriorate

④ Hard to talk

⑤ Lack of knowledge

⑥ Others

2-6. Are you willing to participate in forest healing program education for cancer survivors?

① Yes ② No (→ go to Q 2-7)

2-7. Reasons for not participating in education

① Don't have time ② Not interested ③ I don't think it will help

④ Lack of support ⑤ Others

Section III. Knowledge level and education needs

3-1. Please answer each item regarding your knowledge of cancer survivors and educational needs.-

Domains	Items	Knowledge level				Perceived importance			
		not at all	no well	well	very well	Not at all	A little necessary	necessary	Very necessary
Cancer survivors' overview	Status for cancer survivors	①	②	③	④	①	②	③	④
	Cancer treatment	①	②	③	④	①	②	③	④
	Effective communication with cancer survivors	①	②	③	④	①	②	③	④
	Health status screening method	①	②	③	④	①	②	③	④
Emotional problems	Depression/sadness	①	②	③	④	①	②	③	④
	Fear/worry	①	②	③	④	①	②	③	④
	Nervousness/irritability	①	②	③	④	①	②	③	④
	Loss of motivation	①	②	③	④	①	②	③	④
Physical problems	Change in appearance (hair loss, skin color)	①	②	③	④	①	②	③	④
	Diet (weight/intake change)	①	②	③	④	①	②	③	④
	Fatigue	①	②	③	④	①	②	③	④
	Indigestion (nausea)	①	②	③	④	①	②	③	④
	Memory/reduced concentration	①	②	③	④	①	②	③	④
	Pain	①	②	③	④	①	②	③	④
	Sleep disorder	①	②	③	④	①	②	③	④
	Numbness of limbs	①	②	③	④	①	②	③	④
Spiritual problems	Anxiety about recurrence/death	①	②	③	④	①	②	③	④
	Worries about the meaning of life	①	②	③	④	①	②	③	④
Functional problems	Rasing Children	①	②	③	④	①	②	③	④
	Economic problems	①	②	③	④	①	②	③	④
	Work/school	①	②	③	④	①	②	③	④

- Thank you for your participating-

File S1. Developed questionnaire on educational needs of forest healing instructors related to programs for cancer survivors
